# Current Understanding of West Nile Virus Clinical Manifestations, Immune Responses, Neuroinvasion, and Immunotherapeutic Implications

**DOI:** 10.3390/pathogens8040193

**Published:** 2019-10-16

**Authors:** Fengwei Bai, E. Ashley Thompson, Parminder J. S. Vig, A. Arturo Leis

**Affiliations:** 1 Department of Cell and Molecular Biology, University of Southern Mississippi, Hattiesburg, MS 39406, USA; elizabeth.a.thompson@usm.edu; 2 Departments of Neurology, University of Mississippi Medical Center, Jackson, MS 39216, USA; pvig@umc.edu; 3 Methodist Rehabilitation Center, Jackson, MS 39216, USA; aleis@mmrcrehab.org

**Keywords:** West Nile virus, immune response, neuroinvasion, clinical manifestation, therapeutic implication

## Abstract

West Nile virus (WNV) is the most common mosquito-borne virus in North America. WNV-associated neuroinvasive disease affects all ages, although elderly and immunocompromised individuals are particularly at risk. WNV neuroinvasive disease has killed over 2300 Americans since WNV entered into the United States in the New York City outbreak of 1999. Despite 20 years of intensive laboratory and clinical research, there are still no approved vaccines or antivirals available for human use. However, rapid progress has been made in both understanding the pathogenesis of WNV and treatment in clinical practices. This review summarizes our current understanding of WNV infection in terms of human clinical manifestations, host immune responses, neuroinvasion, and therapeutic interventions.

## 1. Introduction to West Nile virus

West Nile virus (WNV) belongs to the flavivirus genus in the family of Flaviviridae with other globally important viral pathogens. Flaviviruses enter host cells through clathrin-mediated endocytosis, which requires help from a variety of host factors, and release their genome from the endosome into the cytoplasm after acidification-induced membrane fusion [1,2,3]. WNV has an approximately 11 kb long, single-stranded, positive sensed RNA genome, which encodes a single open reading frame with terminal 5′ and 3′ UTRs that have a 5′ cap and no poly-A tail, respectively. Using the host cellular machinery, the viral RNA genome trafficks through the cytosol to the endoplasmic reticulum (ER), where the viral RNA is translated as a polyprotein that is then cleaved into ten functional proteins by both cellular and viral proteases. There are three structural proteins (capsid [C], pre-membrane [PrM/M], and envelope [E]), and seven non-structural proteins (NS1, NS2A, NS2B, NS3, NS4A, NS4B, and NS5) [4,5]. However, studies suggest that additional proteins may also be produced through ribosomal frameshifting [6,7]. While non-structural proteins play essential roles in the replication of viral genomic RNA, the structural proteins assemble the virion and mediate host receptor binding and viral entry. With the help of viral and cellular proteins, the positive-sense viral genomic RNA is transcribed into negative-sense RNA serving as a template for the replication of progeny viral genome RNA. The structural proteins assemble the nucleocapsid on membranes in the ER, and bud into the cytoplasm via the Golgi network. The progeny virus travels to the cell surface in an exocytic vesicle and matures as cellular enzymes cleave the prM, resulting in the release of mature virion [8].

WNV is cytolytic and induces apoptosis in a variety of cell types including neurons, resulting in illness in a range of vertebrate hosts including humans, horses, and birds. In humans, infections with WNV are either asymptomatic or can lead to life-threatening neuroinvasive diseases. Despite intensive laboratory and clinical research in the last 20 years, there are still no approved vaccines or antivirals available for human use, and supportive care remains the standard. Here, we review the current understanding of WNV epidemiology, clinical manifestations, protective immune responses, mechanisms of neuroinvasion and neuropathogenesis, and provide immunotherapeutic implications.

## 2. WNV Epidemiology

In nature, WNV transmission is maintained in a cycle between *Culex* mosquitoes, the major vector, and a variety of bird species, the major reservoir hosts. Humans, horses, and other vertebrate animals can be infected with WNV through the bite of an infected mosquito, but are considered as “dead-end hosts” because infection in mammals does not produce a viremia of sufficient magnitude to infect subsequent biting mosquitoes. According to the Centers for Disease Control and Prevention (CDC), most people (80%) infected with WNV do not develop any symptoms, and about 20% infected people develop a fever with other symptoms such as headache, body aches, joint pains, vomiting, diarrhea, or rash. About 1 in 150 people who are infected with WNV develop a severe illness affecting the central nervous system (CNS), such as encephalitis and meningitis. The virus became recognized as a cause of human meningitis or encephalitis in elderly patients during an outbreak in Israel in 1957 [9], although the CNS involvement in middle-aged and younger subjects remained unusual [10]. During the 1980s and 1990s, there were major outbreaks in Africa, Middle East, Europe, and Russia, although the Romania epidemic in 1996 marked the geographic transition of WNV epidemics from rural areas to urban industrialized areas [11]. In 1999, WNV gained entry into North America in New York City [12], and within three years spread to most of the continental U.S. and the neighboring countries in North America [13]. Phylogenetic analysis of WNV isolated from the U.S. indicates that a single conserved amino acid change in the envelope gene (V159A) is shared by most of the strains isolated since 2002, but is also found in old-world strains of WNV [14]. This genotype has effectively displaced the genotype originally introduced to the U.S. [14]. WNV has now spread across six of seven continents, including Africa, Asia, Europe, Australia (subtype Kunjin), North America, and South America. It is considered one of the most important causative agents of human viral encephalitis worldwide. Since 1999, WNV has been estimated to cause more than 6 million human infections [13], with over 24,000 neurological disease cases and 2300 deaths in the U.S. In addition, in 2018, a large outbreak occurred in Europe involving over 2000 human cases in 15 countries [15]. Therefore, there is an urgent need to understand in-depth the pathogenesis of WNV and develop specific treatment strategies.

## 3. Clinical Manifestations of WNV Infection

*WNV fever and neuroinvasive disease:* WNV infection in humans can result in a spectrum of diseases ranging from a febrile illness classified by the CDC as WNV fever to severe neuroinvasive disease classified as meningitis, encephalitis, or acute flaccid paralysis (poliomyelitis variant) [16]. Before 1996, WNV was primarily known to cause febrile illness, now classified by the CDC as WNV fever and often described as a “summer flu” associated with fever, chills, malaise, headache, backache, myalgias, arthralgias, gastrointestinal symptoms (nausea, vomiting, or diarrhea), and maculopapular rash. By definition, neurological symptoms (neuroinvasion) do not occur in WNV fever patients and most recover completely, but fatigue and weakness can persist for weeks or months [16]. Since 2002, approximately half of reported cases have been classified as WNV fever and half as neuroinvasive disease. For example, in the WNV epidemic of 2012, of the 5387 reported WNV cases nationwide, 2734 (51%) were classified as neuroinvasive disease cases and 2653 (49%) as non-neuroinvasive disease cases [16]. Neuroinvasive disease cases present with more severe symptoms, including encephalitis, meningitis or acute flaccid paralysis [16]. Moreover, WNV infection has also been indicated to cause longitudinally extensive transverse myelitis [17], cognitive-behavioral impairment and irreversible sensorineural deafness [18], and kidney diseases [19]. Symptoms of neuroinvasive disease include high fever, worsening headache, neck stiffness (nuchal rigidity), confusion, stupor, tremors, seizures, muscle weakness or paralysis, and focal neurological deficits. About 1 in 10 people who develop severe CNS illness die [16]. In recent years, severe neurological illness has been reported much more frequently, together with neuromuscular manifestations.

*Neuromuscular manifestations and WNV poliomyelitis:* In the 1999 New York City outbreak, more than 50% of patients with confirmed WNV encephalitis had severe muscle weakness as a cardinal sign [12], and there were several case series that attributed neuromuscular complications, particularly acute flaccid paralysis, to peripheral nerve etiologies, namely Guillain–Barré syndrome (GBS), motor axonopathy, or severe axonal polyneuropathy [12,20,21]. In the 2002 and 2003 WNV epidemics, neuromuscular manifestations were a well-recognized feature associated with increased morbidity and mortality [22,23,24]. Neuromuscular manifestations are now recognized as a prominent feature in patients with WNV neuroinvasive disease [25].

Most physicians who are actively involved in WNV clinical research now accept a poliomyelitis to be the most common cause of WNV-associated acute flaccid paralysis. Patients with WNV poliomyelitis commonly have associated signs of meningitis, encephalitis, or respiratory distress from involvement of spinal motor neurons supplying the phrenic nerves to the diaphragm, however acute flaccid paralysis also may occur in the absence of fever or meningoencephalitis [24,26,27]. Moreover, cases of WNV-poliomyelitis frequently occur in the absence of acute flaccid paralysis, since the current CDC classification recognizes only the most severe cases associated with flaccid paralysis and fails to include poliomyelitis cases with lesser degrees of motor neuron loss that do not produce profound flaccid weakness (Leis, personal observation). Prognosis for recovery of function is dependent on the degree of motor neuron loss; limbs with absent motor responses on nerve conduction studies and no voluntary muscle activation on needle electromyogram (EMG) have a poorer long-term prognosis, while those with relatively preserved motor responses and some voluntary activity have a more favorable outcome [25,28]. Although the anterior horns are the major site of spinal cord pathology, autopsy series show that pathologic changes may not be limited to spinal cord gray matter. Focal inflammatory changes may extend into the adjacent white matter, which may explain the infrequent occurrence of WNV-associated transverse myelitis with clinical involvement of spinal sensory and motor pathways [29]. In addition, neuronophagia, neuronal disappearance, and pathologic alterations have been described in dorsal root ganglia and sympathetic ganglia [29]. The involvement of dorsal root ganglia may explain some of the sensory deficits and reduced sensory nerve action potentials on electrodiagnostic testing that occasionally are reported in patients with WNV infection [24]. However, sensory loss attributed to WNV has not been a prominent clinical finding.

*WNV infection and autonomic nervous system:* To date, there are very few reports on WNV-related autonomic nervous system dysfunction or the role that dysautonomia plays in the morbidity and mortality of human WNV infection. Yet, symptoms of dysautonomia are commonly observed during the acute WNV infection and in WNV survivors recovered from the acute illness. In one study of 44 WNV patients that reported prolonged (> 6 months) post-infectious symptoms, gastrointestinal symptoms (abdominal pain, bloating or diarrhea) were reported by 21 (48%), night sweats in 20 (45%), increased anxiety in 14 (32%) and irregular heartbeat in 9 (20%) [30]. In our initial series of 54 WNV patients from 2002 to 2006 who had extensive electrodiagnostic evaluation and four additional autopsies, 12 (22%) had clinical findings of dysautonomia and autonomic instability (cardiac dysrhythmias, marked fluctuations in blood pressure, gastrointestinal complications including gastroparesis) or pathologic alterations in sympathetic ganglia or brainstem [25]. Post-mortem disappearance of neurons in the sympathetic ganglia offers a plausible explanation for the cardiac instability observed in some human cases [29]. Labile vital signs, hypotension, and potentially lethal cardiac arrhythmias have also been observed by other clinicians [31,32]. In rodents, WNV can lead to autonomic dysfunction by infecting neurons controlling gastrointestinal and cardiac function. In infected hamsters with distension of the stomach and intestines, myenteric neurons that innervate the intestines and neurons in the brainstem were identified to be infected with WNV [33]. The authors concluded that that infected neurons controlling autonomic function were the cause of GI dysfunction in WNV-infected hamsters. Dysautonomia was also noted on electrocardiograms and on measures of heart rate variability in WNV-infected hamsters and cardiac histopathology was identified in the sinoatrial (SA) and atrioventricular (AV) nodes [33]. WNV infection of hamsters has also been reported to cause electrophysiologic suppression of the diaphragm either directly by producing neuronal injury in the brainstem and cervical spinal cord or indirectly by altering vagal afferent function [34], although subsequent investigations suggested that respiratory deficits primarily may be due to loss of lower motor neurons in the cervical cord [35]. Bladder dysfunction and changes in sweating, including hyperhidrosis, anhidrosis and segmental changes in sweating, have also been observed in patients with WNV infection [36]. Hence, both human and animal data suggest that WNV can cause dysautonomia, supporting the concept that the neurotropism of WNV may extend to autonomic neurons in the CNS and autonomic ganglia.

## 4. Protective Immune Responses to WNV Infection

### 4.1. Innate Immunity

The innate immune system serves as the first line of host defense against infections. It detects viral infection through the recognition of pathogen-associated molecular patterns (PAMPs) by pathogen-recognition receptors (PRRs). There are three distinct classes of PRRs in recognizing viruses, including Toll-like receptors (TLRs), retinoic-acid-inducible gene I (RIG-I)-like receptors (RLRs), and NOD-like receptors (NLRs). While TLRs are membrane-associated sensors, either on the cell membrane or in the endosome, RLRs and NLRs detect viruses in the cytoplasm. TLRs recognize viral nucleic acids and other viral components, such as TLR3 and TLR7/8 recognizing double-stranded RNA (dsRNA) and single-stranded RNA (ssRNA) respectively, to trigger the production of interferon I (FN) and other cytokines. WNV has an ssRNA genome but makes dsRNA intermediates while its genomic RNA is replicating, and thus can be recognized by TLR3 [37]. The signaling of TLR3 in mice has been shown to facilitate WNV infection by increasing the permeability of the blood-brain-barrier (BBB), while another report suggested that TLR3 might protect mice from WNV infection [38,39,40]. Our research has demonstrated that TLR7 signaling plays a protective role against WNV infection in mouse models by triggering IL-23 mediated immune cell infiltration into the CNS [41]. Human TLR8 can recognize viral ssRNA, but mouse TLR8 was described as nonfunctional, which may be due to a deletion of 5 amino acids in the leucine-rich repeat ectodomain of TLR8 in mice [42]. Although TLR8 may not directly sense WNV RNA, it interacts with suppressor of cytokine signaling (SOCS)-1 and restrains TLR7 mediated antiviral immunity in mice [43].

The RLR family of cytosolic RNA helicases, which include retinoic acid-inducible gene (RIG-I), melanoma differentiation-associated gene 5 (MDA5) and laboratory of genetics and physiology gene 2 (LGP2), are expressed basally at low levels in most tissues and induced by type I (α/β) IFNs. While short dsRNA and 5′-triphosphate RNA are ligands for RIG-I, MDA5 mainly recognizes long dsRNA [44,45]. Both RIG-I and MDA5 encode two amino-terminal tandem caspase activation recruitment domains (CARDs) that recruit mitochondrial antiviral signaling (MAVS, also known as IPS-1, VISA, or Cardif), upon binding to viral RNA, activating IRF3/7 and NF-κB, leading to the production of type I IFNs and antiviral responses. In contrast, LGP2 (encoded by Dhx58) has a high RNA binding affinity and can recognize diverse dsRNAs, regardless of the presence of 5’-triphosphate or RNA length [46]. Both negative and positive regulatory roles have been reported for LGP2 in antiviral immunity [47,48,49,50,51,52]. LGP2 has been shown to promote an essential prosurvival signal in response to antigen stimulation to confer CD8^+^ T cell-number expansion and effector functions against WNV and other RNA viruses [53].

Mammalian cell PRRs recognize the PAMPs of WNV and induce type I IFN productions during the early stages of WNV infection. Experimental mice with genetic defects in the receptor for IFN-α/β (IFN-α/βR^−/−^ mice with 129Sv/Ev and C57BL/6J background) show markedly enhanced viral accumulation in various tissues, leading to rapid lethality [54]. The induced IFN restricts WNV viral replication, however, WNV has evolved to counter IFN function at multiple steps of the induction and signaling cascade [39,55,56,57,58,59]. For example, NS proteins, such as NS2A, NS3, NS4A, NS4B, and NS5 have been shown to inhibit type I IFN production or signaling [55,60,61,62,63]. In addition, WNV is resistant to the antiviral effects of IFN in cell culture once infection is established, which may explain the relatively narrow therapeutic window for IFN administration that has been observed in animal models or humans infected with WNV [64].

*Dendritic cell and macrophage:* Mosquito transmitted WNV infection begins in the skin, and the virus replicates initially in keratinocytes and Langerhans cells (LCs), skin resident dendritic cells (DCs). After being activated by the WNV antigens, LCs can migrate to the draining lymph node, leading to T-cell priming. In a murine model of dermal WNV infection, bone marrow-derived monocytes infiltrate the skin, cluster near infected fibroblasts and then differentiate into DCs [65]. It has been shown that human monocyte-derived DCs are permissive to WNV infection, and secret high levels of type I IFN but low levels of pro-inflammatory cytokines, such as IL-12, IL-23, IL-18 or IL-10 in response to infection [66,67,68,69]. Interestingly, signaling of type I IFN has been shown to decrease in DCs from aged donors infected with WNV, which could partially explain the more severe WNV infections in elderly patients [67]. However, type I IFN produced from DCs may fail to activate the proinflammatory function of iNKTs, a type of natural killer T cell specifically contributing to antiviral response [66]. Type I IFN may also diminish DC maturation, reduce production of IL-1β, and weaken antiviral T cell immunity in AXL receptor tyrosine kinase deficient (*Axl^−/−^*) mice during WNV and influenza infection [70]. Recent studies showed that IL-1β, IFN regulatory factors (IRF) 3, 5 and 7 are important to induce DC-mediated antivirus IFN responses against WNV infection [71,72,73].

Besides DCs, macrophages as specialized phagocytes and antigen-presenting cells are early responders to control initial WNV replication and present the viral antigens to T cells. Macrophages efficiently ingest WNV through receptor-mediated endocytosis, then become activated to produce a large number of antiviral chemokines and cytokines, such as CXCL1, CXCL2, CXCL10, TNF-α, IL-6, IL-8, IL-1β and type I IFNs. These molecules are critical for the restriction of WNV replication and the recruitment of more innate immune cells to the site of infection [41]. The role of macrophages in resistance to WNV infection has been shown in mice depleted of macrophages, which exhibited higher and extended viremia and accelerated development of encephalitis and death [74]. However, excessive cytokine production and inflammation may increase permeability of the BBB, promoting viral infection of the CNS and severe neurological disease [40]. Microglial cells, the resident macrophages of the CNS, become activated and produce proinflammatory molecules upon WNV infection [40,41]. Recently, it has been shown that defective WNV replication in Peli1 (an E3 ubiquitin ligase)-deficient microglia and neurons directly contributes to attenuated neuroinflammation in the CNS and ultimately increases host resistance to lethal encephalitis [75].

*Neutrophil:* Neutrophils are the most abundant leukocytes in human blood and the first immune cells to arrive at the site of infection. In combating microbial pathogens, neutrophils can engulf pathogens, produce reactive oxygen and nitrogen species, release granules containing proteolytic enzymes and antimicrobial peptides, and extrude neutrophil extracellular traps (NETs) [76]. However, neutrophils have been shown to play complex roles in the pathogenesis of WNV. Neutrophils can serve as reservoirs for WNV replication and dissemination in the early stages of infection but contribute to WNV clearance later in the infection process [77]. In addition, neutrophils have also been documented to facilitate WNV neuroinvasion via “Trojan horse” transport [78,79].

*Natural Killer (NK) cell:* NK cells are important innate immune cells to control viral infection and cancer. NK cells are able to kill virally infected cells by inducing apoptosis of the target cells that are missing major-histocompatibility complex (MHC) class I by releasing cytotoxic granules containing perforin and granzymes as well as producing inflammatory cytokines that limit infection [80]. However, the reported roles of NK cells in controlling WNV infection in mice are complex. Although NK cells can be activated and recruited in the CNS following WNV infection in mice, and they have been shown to lyse WNV-infected cell lines in vitro suggesting a protective role, NK cells seem to fail to kill WNV-infected mouse astrocytes [81]. In addition, depletion of NK cells from circulation of mice either by using an antibody against NK1.1 or in the Ly49a transgenic strain of mice also failed to alter the susceptibility to WNV infection, suggesting NK cells may not have a primary role in controlling WNV infection and disease in mice [82]. In contrast, human NK cells can be activated and able to inhibit WNV infection of Vero cells through both cytolytic and noncytolytic activities [83]. Specific subsets of human NK cells have been shown to mount a robust response to WNV infection, resulting in cytolytic activity, cytokine secretion, and chemokine secretion in vitro [84]. Some flaviviruses including WNV, have developed mechanisms to evade the NK cell response, and the lack of NK protection in mice may be due to WNV suppressing or bypassing the NK response. Therefore, it is reasonable to predict that blocking the viral evasion process and reinforcing NK cell function might lead to improved control and accelerated clearance of the viral infection [83]. The detailed roles of NK cells in the pathogenesis of WNV need to be further investigated.

*Gamma-Delta T cell:* γδ T cells are small subsets of CD3^+^ T cells that respond rapidly to microbial antigens without requiring antigen processing and MHC presentation of peptide epitopes and produce pro-inflammatory cytokines IFN-γ, TNF-α and IL-17. γδ T cells have been documented to play unique roles in many microbial infections, autoimmune diseases, allergies, and immunoregulation. Both protective and pathogenic roles of γδ T cells have been indicated during WNV infection. In mouse models, both γδ T cell-deficient (TCRδ^−/−^) mice and mice depleted of Vγ1+ cells, a subset of γδ T cells, had elevated viremia and more severe encephalitis compared to the wild-type (WT) control mice, which may be due to the reduced IFN-γ production in these animals [85,86,87]. In addition to IFN-γ, γδ T cells have also been suggested to contribute to regulate intracellular perforin levels against WNV in the periphery αβ T cells [86]. In contrast, Vγ4+ cells, another major subpopulation of peripheral γδ T cells have been implied to contribute to WNV pathogenesis in mice. Depletion of Vγ4^+^ cells in mice resulted in lower levels of viremia, encephalitis, and mortality rates following WNV infection. The pathogenic effects of Vγ4+ cells are mediated via the production of proinflammatory and regulatory cytokines, including TNF-α, IL-10, and TGF-β. TNF-α has been shown to compromise the integrity of the BBB and facilitate WNV entry into the brain [40]. Anti-inflammatory cytokine IL-10 has been shown to play a negative role in controlling WNV infection in mice [88]. Vγ4^+^ cell-depleted mice had reduced levels of TNF-α and IL-10, accompanied by a decreased viral load in the brain and a lower mortality rate to WNV encephalitis [89]. In addition, Vγ4+ cells negatively regulate Vγ1^+^ T cell responses via the production of TGF-β during WNV infection [89].

### 4.2. Complement

The complement system consists of at least 30 serum and cell surface proteins that interact with each other through a cascade of enzymatic activation and inhibition, leading to the elimination of pathogens, while protecting the self from subsequent damage. While activation of the complement system can be initiated by three different pathways, i.e., the classical pathway, the lectin pathway, and the alternative pathway, all the three pathways share a common amplification cascade of enzymatic reaction, leading to the formation of C5b-9 membrane attack complex (MAC). The classical pathway is activated by C1q binding to antigen-antibody complexes on the surfaces of pathogens. The lectin pathway is initiated by binding of mannose-binding lectin (MBL) to microbial surface, resulting in activation MBL-associated serine proteases (MASPs). The alternative pathway is constitutively active at low levels through the spontaneous hydrolysis of C3. The complement system has a protective role in controlling WNV infection. WNV infection in mice deficient in complement receptor 1 (CR1) and CR2 developed increased CNS virus burdens, and were vulnerable to lethal infection at a low dose of WNV, and had significant deficits in IgM and IgG [90]. In addition, genetic deficiencies in C1q, C4, factor B, or factor D all resulted in increased mortality in mice following WNV infection. Furthermore, the lack of complement functions might also result in the deficiency of B and T cell responses to WNV [91]. MBL recognizes N-linked glycans on the structural proteins of WNV and DENV, resulting in neutralization through a C3- and C4-dependent mechanism that utilizes both the canonical and bypass lectin activation pathways [92]. Soluble and cell-surface-associated WNV NS1 has been shown to bind to and recruit factor H, resulting in decreased complement activation in solution and attenuated deposition of C3 fragments and MAC on the cell surface [93]. NS1 attenuates classical and lectin pathway activation by directly interacting with C4 [94,95]. The complement component C1q increases the potency of antibodies against WNV by modulating the stoichiometric requirements for neutralization [96]. However, another study reported that despite mice deficient in MBL-A and MBL-C or MASP-2 were more vulnerable to WNV infection than WT mice, they had comparable kinetics of viral burden in the CNS and B and T cell immune responses, suggesting that lectin pathway might have important yet subordinate functions in protecting against WNV infection [97]. In addition, using C5-depleted or -deficient human or mouse sera, one study suggested that C5 might not either contribute to protection against WNV pathogenesis or augment the neutralizing efficacy of complement-fixing anti-WNV neutralizing antibodies in mice [98].

### 4.3. Adaptive Humoral Immunity

Adaptive immunity is composed of humoral (B cell)- and cellular (T cell)-based immune responses. The humoral immune response plays a key role in the pathogenesis of WNV. Antibody and B cells are essential in the defense against WNV infection in that mice deficient in B cells and antibody (μMT mice) developed increased serum and CNS viral burdens and were vulnerable to lethal infection [99]. Prophylactic and therapeutic transfer of human IgG or mouse serum delayed or protected WT, μMT and RAG1 (T and B cell-deficient) mice from WNV infection. Antibody by itself did not eliminate viral reservoirs in host tissues, suggesting an intact cellular immune response may be required for viral clearance [100]. Furthermore, sIgM^−/−^ mice, which are deficient in the production of secreted IgM but capable of expressing surface IgM and secreting other antibody isotypes, are vulnerable to lethal WNV infection [101]. These results demonstrated that the early anti-WNV IgM in the course of WNV infection limits viremia and dissemination into the CNS. The innate immune responses are required to activate B cell response and antibody production. A study showed that mice deficient in MyD88, the essential adaptor for the signaling of the majority of TLRs except TLR3, significantly diminished B cell response by impairing B cell activation, development, and generation of long-lived plasma cells and memory B cells. In contrast, TLR3 deficiency had more effects on the maintenance of germinal centers and the development of long-lived plasma cells, whereas, differentiation of memory B cells was unaffected [102]. Signaling through type I IFN-α/β receptor has also been shown to be required for B cell activation in the draining lymph nodes, but not the spleen [103]. In line with this, mice deficient in interferon-induced transmembrane protein, Ifitm3, infected with WNV showed decreases in the total number of B cells, CD4^+^ T cells, and antigen-specific CD8^+^ T cells and increases in viral burden in the peripheral organs and CNS tissues [104]. However, mice deficient in interferon regulatory factor 5 (*Irf5^−/−^*) infected with WNV exhibited small differences in the type I IFN response also resulted in reduced IgM and IgG secretion that was associated with fewer antigen-specific memory B cells and long-lived plasma cells, which may relate to the reduced proinflammatory cytokine production in these mice [105]. Furthermore, B cell and humoral responses to WNV infection have been suggested to be regulated by many factors related to DCs, the complement system, CD4^+^ T cells and other immune factors [91,106,107,108,109,110].

*CD8^+^ T cell:* CD8^+^ T cells control viral infections by triggering apoptosis of infected cells through perforin- or Fas ligand-dependent pathways or producing antiviral cytokines (e.g., IFN-γ and TNF-α). CD8^+^ T cells are essential in protecting from WNV infection. Early studies using rag-deficient mice that lack all T cells and B cells, CD8^+^ T cell and MHC class Ia-deficient mice demonstrated a protective role in clearing WNV infection in the CNS and peripheral tissues [100,111]. In addition, WNV infection of mice deficient in perforin, fas ligand and TRIAL that are the vital elements for CD8^+^ T cell killing resulted in the increased mortality following infection [82,112,113]. Furthermore, WNV-infected mice deficient in chemokine CXCL10 and its receptor CXCR3, which are essential for CD8^+^ T cell chemotaxis to the CNS, especially cerebellum, increased in viral burden in the brain and enhanced morbidity and mortality [114,115]. Polarized expression of CXCL12 and its receptor CXCR4 at the BBB localizes infiltrating mononuclear cells to the perivascular spaces of the CNS microvasculature, therefore limiting their entry into the CNS parenchyma [116]. A study showed that antagonism of CXCR4 significantly improved survival through enhanced intraparenchymal migration of WNV-specific CD8^+^ T cells within the brain [117]. Moreover, enhanced CD8^+^ T cells cytotoxicity through a supplement of IL-17A has been shown to increase the survival of the mice from WNV infection [118]. Collectively, the results from these studies indicated that CD8^+^ T cells play an important protective role in controlling WNV infection. However, studies also showed a potential pathogenic role for CD8^+^ T cells in WNV infection. High doses (10^8^ PFU) of Sarafend strain of WNV infection in *CD8^−/−^* mice lead to the increased survival, while low doses (10^3^ PFU) of infection resulted in more severe infection, indicating that CD8^+^ T cells are involved in both recovery and immunopathology in WNV infection [119]. Additionally, when mice lacking the interferon-stimulated gene Ifit1 were depleted with CD8 antibody, they survived better than the isotype control mice following a mutant WNV (E218A) infection, in which NS5 lacks 2′-O methyltransferase activity, indicating CD8^+^ T cells contribute to mortality [120]. Therefore, while CD8 T cells are required for control of WNV infection, they may also contribute to immunopathogenesis in some situations.

*CD4^+^ T cell:* CD4^+^ T cells play a central role in the adaptive immune system in that they provide help to B cell for antibody production and CD8^+^ T cells to kill the cells infected with intracellular pathogens. CD4^+^ T cells have been documented to have both indirect and direct roles in defense against WNV infection. Infection of mice that lack CD4^+^ T cells either by antibody depletion or genetic deficiencies in CD4^+^ T cells and MHC class II resulted in a prolonged WNV infection in the CNS that culminated in uniform lethality by 50 days after infection. CD4 deficient mice exhibited significant low levels of IgG and CD8^+^ T compared to WT control mice, indicating an indirect protective role of CD4^+^ T cell during primary WNV infection by sustaining antibody production and effector CD8^+^ T cells that allow rapid viral clearance [110]. In addition, evidence suggests CD4^+^ T cells may also play a direct role in limiting WNV replication by producing IFN-γ, IL-2 and granzyme B, and lysing target cells both in vitro and in vivo [121,122,123]. Regulatory T cell (Treg) is a subset of CD4^+^ T cells that can suppress effector T cells to prevent or control reactivity to self or non-self antigens, and to blunt inflammation. However, studies in human and mouse models have shown that Tregs appeared to play a protective role in limiting WNV disease. Tregs expanded significantly in the peripheral after WNV infection in both humans and mice. In humans, the Treg frequencies were lower in the symptomatic donors than the asymptomatic donors. In parallel prospective studies in mice, symptomatic WNV-infected mice also developed lower Treg frequencies compared with asymptomatic mice after infection and Treg-deficient mice developed lethal WNV infection at a higher rate than the controls [124]. In addition, Treg-dependent production of TGF-β results in increased expression of CD103 on CD8^+^ T cells in the spleen and brain, indicating that Tregs are essential to maintain the resident memory T cells post-WNV infection [125]. Furthermore, innate immune signaling TLR7 seemed to positively regulate Treg expansion, while intrinsic MAVS signaling, the signaling adaptor for RNA helicases such as RIG-I, was dispensable for Treg proliferation and suppressive capacity during WNV infection in mice [126,127].

## 5. WNV Neuroinvasion and Neuropathogenesis

**Figure 1:**
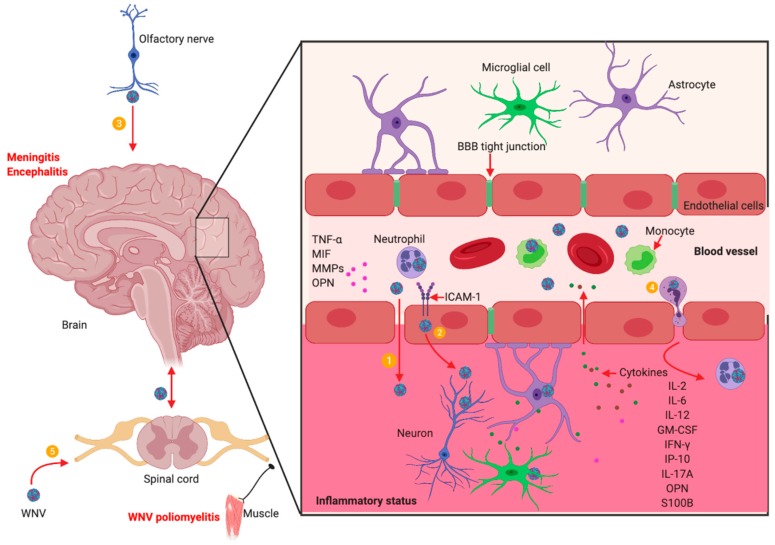
West Nile virus (WNV) neuroinvasion and neuropathogenesis.

*Neuroinvasion:* WNV can enter the CNS and cause neuroinvasive diseases with possible long-term consequences [128,129]. Although the detailed mechanisms are still not well understood, WNV has been suggested to enter the CNS through multiple possible pathways, including flow through the blood-brain-barrier (BBB) tight junctions, direct infection of endothelial cells in the cerebral microvasculature, infection of olfactory neurons, infected leukocytes that “Trojan horse” WNV to the CNS, and/or direct axonal retrograde transport from infected peripheral nerves [87,91,92,93,94,95,96,97] (Figure 1).

WNV replication in the peripheral organs and the blood cells, such as neutrophils and monocytes, generates viremia in the blood circulation that may lead to infection in the CNS. WNV infection in the CNS can result in meningitis, encephalitis, and acute flaccid paralysis, including WNV poliomyelitis. The possible mechanisms by which WNV enters the CNS include: ① WNV infection induces the expression of TNF-α, MIF, MMP9, ICAM-1 and Opn, which directly or indirectly increase the permeability of the BBB allowing the virus to penetrate to the CNS; ② WNV may infect endothelial cells in the cerebral microvasculature, from which progeny viruses may be released into the CNS; ③ WNV may enter the CNS from infected olfactory bulbs via olfactory neurons; ④ WNV-infected leukocytes, such as neutrophils via a “Trojan horse” transport of WNV to the CNS; and ⑤ WNV may be transported to the CNS through the infected peripheral nerves. In the CNS, WNV may infect neurons, microglia, and astrocytes producing cytokines and chemokines and leading to inflammation, neuron apoptosis and necrosis. Some molecules including IL-2, IL-6, IL-12, GM-CSF, IFN-γ, IP-10, S100B, IL-17A and OPN, remain persistently elevated in the blood for months after clearance of WNV from the body, which can lead to a post-infectious pro-inflammatory state that may promote autoimmune diseases, such as myasthenia gravis. The illustration was created in Biorender.com.

The BBB, composed of endothelial cell tight junctions and astrocyte extensions, poses an obstacle for the entry of pathogens into the brain [130]. However, neurotropic viruses are capable of crossing the BBB and causing inflammation and neuronal death in the CNS. In mice, WNV induces systemic infection and then crosses the BBB, leading to neuronal infection and damage [40,131,132]. It has been shown that TNF-α receptor-1 signaling and the expression of matrix metalloproteinase 9 (MMP9), intercellular adhesion molecule 1 (ICAM-1, or CD54) and macrophage inhibitory factor (MIF) are vital for the transient BBB permeabilization upon WNV infection [40,131,132,133]. In addition, a study on Venezuelan equine encephalitis virus (VEEV, a mosquito-transmitted alphavirus) infection indicated that the virus initially enters the CNS through the olfactory pathway and initiates viral replication in the brain, which induces the opening of the BBB, allowing a second wave of virus from the periphery to enter the brain [134]. While the initial site of WNV entry into the CNS is still debatable, the rostral-caudal spread of WNV antigen from the olfactory bulb to the remaining structures of the brain has been reported [135,136]. Our results in mice also support this notion because olfactory bulbs have been observed to have more WNV antigens than other brain regions by immunostaining in the early time points of CNS infection [41,88,137], suggesting olfactory bulbs are a possible route of WNV entry into the CNS bypassing the BBB. In contrast, WNV antigen localizing to the olfactory lobes in horses and humans is rare [138,139,140], and viral antigen is primarily seen in mid to hindbrain and spinal cord [139,140]. These non-comparable results may be due to postmortem delays or inconsistent sampling of the olfactory bulb in these hosts [138]. Moreover, infected T cells and macrophages have been suggested carriers of WNV into the CNS [141,142,143], however, our results demonstrated that WNV replicates more efficiently in human and mouse polymorphonuclear neutrophils (PMNs) than in macrophages [77]. In mice, PMNs are recruited into the brain at an early stage of WNV infection [144]. In addition, clinical data also showed that PMNs were the predominant infiltrating leukocytes into the CNS of patients at the early stage of WNV neuroinvasive disease [145,146]. Our recent results show that WNV-infected neutrophils contribute to the viral burden in the mouse brain, suggesting neutrophils could play a major “Trojan horse” role in transporting WNV into the CNS [78]. However, it is possible that some of the neutrophils were infected after they arrived in the CNS.

*Neuropathogenesis:* WNV-mediated death of infected cells can occur by either necrosis or apoptosis [128,147,148]. In cultured Vero cells, a high multiplicity of infection (M.O.I) is followed by necrosis; whereas a low M.O.I results in mitochondrial-dependent apoptosis [147]. Studies have shown that both capsid protein and activation of immune cells such as CD8^+^ T cells can induce apoptosis in neurons through the activation of mitochondrial/caspase-9 and caspase-3 pathways during WNV infection [82,113,147]. In WNV-infected mice, apoptotic neurons are prominent in the spinal cord but rare in the brain, suggesting that the mechanisms of WNV induced cell death may differ within specific regions of the CNS [128]. In the CNS, WNV infects neurons, basal ganglia, brainstem and spinal cord [140], while in cell culture WNV infects both neurons and astrocytes, although with differing kinetics [149]. Infection of microglial cells results in the release of proinflammatory cytokines (IL-6, TNF-α), and chemokines (CXCL10, CCL2, CCL5) [150]. Infected neurons and astrocytes also produce chemokines including CXCL10 and CCL5, which may play a critical role in the recruitment of virus-specific T cells into the CNS [149,151,152].

Histopathologic analysis of brain and spinal cord tissues from patients with neuroinvasive diseases showed perivascular inflammation, microglial nodules, neuronophagia, and variable necrosis and neuronal loss [29]. These changes were prominent in deep nuclei of the brain and anterior horns of the spinal cord. Focal demyelination, gliosis, and occasional perivascular infiltrates were also observed among patients with prolonged clinical courses [153]. Examination of sera and cerebrospinal fluid (CSF) from WNV patients who had symptoms between 2–18 days showed elevated levels of glial fibrillary acidic proteins (GFAP)-SM 126 and S100B, suggesting astroglial activation [154,155]. Severe degenerative changes in the spinal nerves and to a lesser degree the dorsal root ganglion was also observed in patients who succumbed to neuroinvasive disease [156]. Others have shown that receptor for advanced glycation end products (RAGE) could also be activated by HMGB1 released from WNV infected cells undergoing necrotic cell death [157,158]. The spinal nerves exhibited swollen axons surrounded with a thin layer of myelin sheath as well as degenerating axons with disrupted myelin sheaths. The astrocytic protein S100B co-localized to these myelin layers and showed an overall increase in S100B immunoreactivity in the WNV compared to the control specimens, which may lead to RAGE mediated damage at other unaffected regions [156]. In addition, WNV infection could result in a focal loss of ganglionic neurons in the dorsal root and sympathetic ganglia, which may be severe enough to produce sensory deficits in some WNV patients [29].

*Post-infectious CNS symptoms:* Human acute WNV infection induces a significant upregulation of various proinflammatory proteins and cytokines that may initiate and maintain inflammation. Although induction of neuroinflammation is necessary for defense against many viral infections, including other flaviviruses, it is also recognized as a potential contributor to neuropathogenesis [159,160,161]. Moreover, levels of some cytokines including IL-2, IL-6, IL-12, granulocyte-macrophage colony-stimulating factor (GM-CSF), IFN-γ, IFN-γ induced protein 10 (IP-10), IL-17A and OPN [30,78,118], have been reported to be elevated for months to years following the recovery of acute illness, leading to a post-infectious proinflammatory state that may contribute to long-term neuroinflammation in WNV survivors [30,78,118]. Among a large cohort of participants with a history of WNV infection in Houston, TX, USA, approximately 40% of those who presented with clinical disease continued to experience WNV-related morbidity up to eight years post-infection [162]. Another observational study on the Houston cohort showed that the majority of patients (86%, 30/35) with encephalitis had abnormal neurological exam findings compared with uncomplicated fever (27%, 3/11) and meningitis (36%, 5/14) cases during 1–3 years following WNV infection. At the second assessment between 8–11 years post-infection, 57% (4/7) of West Nile fever, 33% (2/6) of West Nile meningitis, and 36% (5/14) of West Nile encephalitis had developed new neurological complications [163]. Importantly, MRI (magnetic resonance imaging) evaluation of the Houston cohort demonstrated that participants with abnormal neurologic and neurocognitive exam findings showed significant cortical thinning as compared to age- and gender-matched controls, with affected regions primarily occurring in the frontal and limbic cortices. Regional atrophy occurred in the cerebellum, brain stem, thalamus, putamen, and globus pallidus [164]. These MRI findings provide valuable evidence regarding the neurologic outcomes following WNV CNS infection. Post-infectious CNS symptoms included post-exertion fatigue, dizziness, altered sensation, arthralgias, dysautonomia impaired memory, difficulty concentrating, depression, anxiety, sleep disruption, and recurrent headaches [30]. Moreover, numerous WNV patients have developed autoinflammatory or autoimmune-related diseases, including myasthenia gravis (MG) [165,166], suggesting that WNV infection may promote or amplify underlying autoimmunity. Interestingly, RAGE and S100B signaling has been reported to promote the development of experimental autoimmune MG [167] and to play a role in the pathophysiology of human MG [168]. In addition, IL-17A is upregulated in human cases of MG, where it plays a vital role in the pathogenesis of MG with higher levels correlating with greater disease severity and thymic hyperplasia [169]. IL-17-producing CD4^+^ T cells also contribute to a loss of B-cell tolerance in experimental autoimmune MG [170]. These results indicate that over-expression of IL-17 following WNV infection could contribute to the development of MG, although it can be beneficial in clearing WNV by promoting CD8^+^ T cell cytotoxicity [118]. Furthermore, WNV infection has also been suggested to induce other autoimmune diseases, including GBS and other immune-mediated neuropathies [31,171], brachial plexopathies [172], stiff-person syndrome [173], and autoimmune encephalitis [174]. However, more clinical data and experimental evidence are still needed to establish the cause-effective relationship of WNV infection with autoimmune diseases.

## 6. Immunotherapeutic Intervention

Despite the development of four licensed veterinary WNV vaccines, there are still no approved vaccines or antivirals available for human use. Although several clinical trials have been initiated with multiple vaccine candidates, none have proceeded to the final phases of clinical testing or are even close to meeting U.S. Food and Drug Administration (FDA) approval (reviewed in [15]). The general reasons for the lack of a human vaccine may include scientific challenges (mainly in obtaining protective immunity), safety concerns (including potential antibody-dependent enhancement responses to other flavivirus infections), difficulties in clinical study design (due to lack of enough WNV cases for a phase III vaccine efficacy study), and economic considerations [175]. While the development of new candidate vaccines and improvement of vaccination strategies remains a challenging task, clinical researches have reported that administration of intravenous high dose steroids may result in marked clinical improvement in WNV patients who suffer severe neuroinflammation.

Both experimental and clinical evidence suggest that pro-inflammatory mediators may contribute to the pathogenesis of WNV in the CNS. Treatment of infected neuronal cells with antibodies blocking these proinflammatory mediators can result in a significant reduction of WNV-mediated neuronal death in vitro [160,161]. Accumulating clinical evidence also strongly suggests that a prolonged post-infection inflammatory state may promote persistent and recurrent symptoms, delayed neurological deficits, and de novo autoimmune disorders, and amplify preexisting autoimmune diseases long after clearance of the virus. At present, no specific therapy has been approved for human use against these post-infectious CNS symptoms. However, an in-depth understanding of the immune-mediated post-infectious pathogenesis of WNV infection has direct therapeutic implications and treatment strategies that suppress this pathogenic immune cascade may prove particularly beneficial. Indeed, in several case series administration of high dose steroids has been documented to improve the outcomes of patients with WNV neuroinvasive disease. In 14 patients with acute WNV meningoencephalitis, intravenous dexamethasone injection was suggested to play a critical role in shortening the acute phase of WNV disease and hastening patient recovery [176]. In addition, high-dose steroids were also used to successfully treat a patient with WNV-associated acute flaccid paralysis and autoimmune encephalitis [174,177]. Two patients with acute flaccid paralysis and brainstem involvement, including progressive seventh nerve palsies, showed clear improvement in brainstem symptoms and facial paralysis within 24 h of treatment with high-dose intravenous methylprednisolone [25]. A recent report of a ‘Lazarus-like effect’ of high dose corticosteroids in a semi-comatose woman with WNV encephalitis highlights the success of this therapeutic approach [178]. Although the use of steroids in patients with WNV neuroinvasive disease seems counterintuitive, with concern that immunosuppressive effects may promote viremia and worsen outcome, the evidence is compelling that WNV is rapidly cleared by immune responses in immunocompetent patients [179]. Additional evidence that immunosuppression does not worsen outcome comes from large scale trials in dengue, a related flavivirus. A comprehensive review that included 13 studies enrolling 1293 participants on the effectiveness of corticosteroids in the treatment of dengue, found no evidence that administration of high doses of oral or intravenous corticosteroids promoted viremia [180]. Rather, therapeutic benefit was seen in multiple studies that used intravenous delivery of high doses and multiple doses of steroids. In contrast, studies without the administration of repeated high doses of steroids showed limited or no beneficial effect. Such clinical observations provide a compelling argument for the use of high dose steroids and possibly other immunosuppressive drugs in the treatment of WNV infection. The clinical experience warrants appropriate large-scale clinical trials to confirm the therapeutic benefit of corticosteroids and other immunosuppressive drugs in the treatment of WNV disease. This is particularly pertinent in light of the fact that there has been no successful development or marketing of a human WNV vaccine.

## 7. Concluding Remarks

WNV has become a major global health concern in the past 20 years and its geographic range now includes six of seven continents. Basic and clinical research has made great progress in understanding WNV biology, transmission, host immune responses, neuroinvasion, and immunopathogenesis. However, no specific therapy or vaccine has been approved for human use and supportive care remains the standard. Merging the current knowledge of post-infectious immune-mediated pathogenesis is critical to navigating therapeutic implications and treatment strategies that control this immunological cascade have already proven beneficial in clinical patient management. Appropriate clinical trials to confirm this therapeutic benefit are needed. Notwithstanding, future research must still be directed at further understanding of the pathogenesis of WNV infection, both in animal models and humans, that will aid in the development of novel and effective therapeutics and vaccine candidates.
